# The Modified Yale Food Addiction Scale 2.0: Validation Among Non-Clinical and Clinical French-Speaking Samples and Comparison With the Full Yale Food Addiction Scale 2.0

**DOI:** 10.3389/fpsyt.2020.480671

**Published:** 2020-09-08

**Authors:** Paul Brunault, Sylvie Berthoz, Ashley N. Gearhardt, Fabien Gierski, Arthur Kaladjian, Eric Bertin, André Tchernof, Laurent Biertho, Arnaud de Luca, Régis Hankard, Robert Courtois, Nicolas Ballon, Farid Benzerouk, Catherine Bégin

**Affiliations:** ^1^ UMR 1253, iBrain, Université de Tours, Inserm, Tours, France; ^2^ CHRU de Tours, Service d’Addictologie Universitaire, Équipe de Liaison et de Soins en Addictologie, Tours, France; ^3^ Qualipsy EE 1901, Université de Tours, Tours, France; ^4^ Univ. Bordeaux, CNRS, EPHE, INCIA, UMR 5287, Bordeaux, Paris, France; ^5^ Département de Psychiatrie de l’Adolescent et du Jeune Adulte, Institut Mutualiste Montsouris, Paris, France; ^6^ Department of Psychology, University of Michigan, Ann Arbor, MI, United States; ^7^ CHU de Reims, Pôle de Psychiatrie adulte, Hôpital Robert Debré, Reims, France; ^8^ Université Reims Champagne-Ardenne (URCA), laboratoire C2S (EA 6291), Reims, France; ^9^ CHU de Reims, Service d’Endocrinologie-Diabète-Nutrition, Hôpital Robert-Debré, Reims, France; ^10^ Institut de Cardiologie et de Pneumologie de Québec, Université Laval, Québec, QC, Canada; ^11^ CHRU de Tours, Service de Médecine Interne-Nutrition, Tours, France; ^12^ Université de Tours, Inserm, UMR 1069, Tours, France; ^13^ CHRU de Tours, Clinique Psychiatrique Universitaire, CRIAVS, Tours, France; ^14^ École de Psychologie, Université Laval, Québec, QC, Canada

**Keywords:** food addiction, substance-related and addictive disorders, obesity surgery, eating addiction, eating disorders, psychometrics, factor analysis, psychopathology

## Abstract

**Objectives:**

The modified Yale Food Addiction Scale 2.0 (mYFAS 2.0) was designed to assess food addiction using a shorter version than the YFAS 2.0. We lack data about the psychometric properties of the mYFAS 2.0 in patients with obesity, as well as studies comparing the psychometric properties of the mYFAS 2.0 versus the full YFAS 2.0. This study aimed to validate the French-language mYFAS 2.0 in a non-clinical population (study 1, n = 250), to determine the yet unknown psychometric properties of this scale in patients with obesity (study 2, n = 345), and to compare the full YFAS 2.0 and the mYFAS 2.0 in terms of food addiction (FA) prevalence and symptoms detection in both populations.

**Method:**

Study 1 included 250 non-clinical individuals (non-underweight and non-obese persons screened negative for eating disorders). Study 2 included 345 bariatric surgery candidates recruited in three centers (Québec, Canada; Reims and Tours, France). The mYFAS 2.0 structure was investigated using confirmatory factorial analyses with tetrachoric correlations. Convergent validity was tested using the full YFAS 2.0, the Binge Eating Scale (both studies), the revised 18-item Three Factor Eating Questionnaire (study 1), the Beck Depression Inventory (study 2), and the body mass index (BMI; both studies).

**Results:**

The mYFAS 2.0 was unidimensional, and had adequate (study 1: KR-20 = .78) and acceptable (study 2: KR-20 = .73) internal consistency. In study 1, the mYFAS 2.0 had good convergent validity with the YFAS 2.0, BMI, binge eating, cognitive restraint, uncontrolled eating and emotional eating; in study 2, the mYFAS 2.0 had good convergent validity with the YFAS 2.0, binge eating, depression, but not BMI. Participants endorsed fewer symptoms with the mYFAS 2.0 than with the YFAS 2.0; FA prevalences were similar between questionnaires in the non-clinical, but not in the clinical sample. A FA ‘diagnosis’ and risk of binge eating disorder were associated but did not completely overlap.

**Conclusions:**

The mYFAS 2.0 has close psychometric properties to the YFAS 2.0 in non-clinical and clinical samples. However, the use of the mYFAS 2.0 in bariatric surgery candidates might lead to a significant underestimation of FA prevalence and number of FA symptoms.

## Highlights

- The mYFAS 2.0 and full YFAS 2.0 have similar psychometric properties: one-factor structure, acceptable internal consistency, and good convergent validity.
- In the non-clinical population, the mYFAS 2.0 did not differ from the full YFAS 2.0 in terms of FA prevalence, but it underestimated the number of FA symptoms.
- In patients with obesity and seeking surgical treatment, the mYFAS 2.0, when compared to the full YFAS 2.0, underestimated the FA prevalence and number of FA symptoms.

## Introduction

In the modern food environment, some but not all individuals struggle to control their food intake (1). In some cases, like for drug misuse, it has been proposed that the loss of control over some specific foods (i.e., highly palatable/processed foods that are high fat, high refined carbohydrates, and/or high salt) may be conceptualized as an addictive disorder (2–4). The term “food addiction” (FA) was first proposed by Randolph in 1956 in the second part of the 20th century to describe an excessive/compulsive food intake of these foods (5). Although FA is not included in the current international diagnostic classifications as a standalone disorder, some authors have questioned the validity of the concept and/or its assessment. According to some authors, it is not clear whether high FA scores are a marker of disordered eating problems generally or a specific assessment of “FA” (6). On the other hand, some other authors have argued that food and drug addiction share similar features that may reflect common underlying mechanisms, paving the way for better tailor-based treatment for these patients [for an updated review of the opposing positions on the concept of FA, see Fletcher and Kenny (6)]. The recent increases in non-homeostatic eating and diet-induced obesity, in addition to the development of assessment tools to operationalize FA, have also enhanced scientific interest for this topic (7, 8).

The most commonly used measurement to assess the construct of FA is the Yale Food Addiction Scale (YFAS), which was published in 2009 (9). The YFAS, also named original YFAS, alters the DSM-IV-TR criteria for substance use disorder (SUD) to be applicable to eating behaviors. More specifically, Gearhardt and colleagues hypothesized that some people may develop addictive-like eating symptoms towards specific foods high in fat and/or refined carbohydrate (9). The original version of this self-report questionnaire is composed of 25 items, loading on a single factorial solution, which explores seven symptoms of addictive-like eating toward these foods: loss of control (substances taken in larger amount and for a longer period than intended), inability to cut down (persistent desire or repeated unsuccessful attempt to quit), much time spent (much time/activity to obtain, use, and recover), impact on activities (important social, occupational, or recreational activities given up or reduced), withdrawal [(development of physiological and/or psychological symptoms in response to abstinence or decreased use of these foods, including, but not limited to, headaches, fatigue, irritability, nervousness or sadness); consumption of foods to relieve these withdrawal symptoms (10); for more details on the types of symptoms experienced during withdrawal and the proposed mechanisms underlying withdrawal, see Schulte et al. (11) who designed the Highly Processed Food Withdrawal Scale], use despite problems (use continues despite knowledge of adverse consequences), and tolerance (marked increase in amount to experience the same effects, and marked decrease in effects with the same amounts). Additional items assess clinically significant impairment and distress. A few years later, a shortened version of the YFAS was developed for a quicker screening in epidemiologic studies [the mYFAS; (12)] and encompasses nine items: seven are based on the core symptoms of addictive disorders, and two additional items assess clinically significant impairment and distress, respectively.

More recently, in line with the DSM-5 update in addictive disorders criteria [now called, substance-related and addictive disorders (SRAD)], Gearhardt et al. (13) updated the YFAS and mYFAS to ensure that measurements of the construct reflected these changes. The YFAS 2.0 includes 35 items that assess the 11 SRAD (the original seven symptoms plus the addition of intense cravings, use that causes interpersonal problems, inability to fulfill role obligations, and increased risk of physical harm) DSM-5 criteria toward highly palatable foods plus clinically significant impairment or distress. The shortened mYFAS 2.0 version was then derived from the YFAS 2.0 by selecting one item for each of the 11 diagnostic criteria plus two items to assess clinically significant impairment and distress, respectively (14). In order to better clarify the evolution in the different versions of the YFAS, we provide information regarding the different versions of the original YFAS, original mYFAS, YFAS 2.0, and mYFAS 2.0 in **Table 1**. The mYFAS 2.0 and YFAS 2.0 have two scoring methods. First, there is a continuous scoring method that summarizes how many of the 11 SRAD criteria an individual endorsed with respect to the consumption of highly palatable foods. Second, the measurement can be scored to assess a ‘diagnostic’ threshold, which can be met if an individual endorses two or more symptoms plus impairment or distress. Although there is no “FA diagnosis” currently recognized in the international diagnostic classifications, this diagnostic threshold is based on the cutoff for a DSM-5 substance use disorder. For individuals who meet the criteria for an YFAS 2.0 ‘diagnosis’ of FA, severity thresholds are also specified (mild = two to three symptoms plus impairment or distress, moderate = four to five symptoms plus impairment or distress, and severe = six or more symptoms plus impairment or distress).

**Table 1:** Correspondence between the DSM-IV-TR SUD/DSM-5 SRAD criteria presumably assessed and the original YFAS, original mYFAS, YFAS 2.0, and mYFAS 2.0 items.

	Original YFAS	Original mYFAS	YFAS 2.0	mYFAS 2.0
*Prevalence for each food addiction criteria*				
Consumed more than planned	#1 #2 #3	#2	#1 #2 #3	#3
Unable to cut down (=persistent desire or unsuccessful efforts to cut down or control consumption of certain foods)	#4 #22 #24 #25	#4	#4 #25 #31 #32	#32
Great deal of time spent	#5 #6 #7	#5	#5 #6 #7	#5
Important activities given up	#8 #9 #10 #11	#9	#8 #10 #18 #20	#10
Use despite physical/emotional consequences	#19	#19	#22 #23	#22
Tolerance	#20 #21	#21	#24 #26	#24
Withdrawal	#12 #13 #14	#12	#11 #12 #13 #14 #15	#13
Use despite social/interpersonal consequences	–	–	#9 #21 #35	#35
Failure in role obligations	–	–	#19 #27	#19
Use in physically hazardous situations	–	–	#28 #33 #34	#33
Craving	–	–	#29 #30	#29
Impairment or distress in relation to food	#15 #16	#15 #16	#16 #17	#16 #17

original YFAS, Yale Food Addiction Scale (based on DSM-IV-TR criteria for substance use disorders; 25 items); original mYFAS, modified version of the original YFAS (based on DSM-IV-TR criteria for substance use disorders; 9 items); YFAS 2.0, Yale Food Addiction Scale 2.0 (based on the DSM-5 criteria for Substance-related and addictive disorders; 35 items); mYFAS 2.0, modified version of the Yale Food Addiction Scale 2.0 (based on the DSM-5 criteria for substance-related and addictive disorders; 13 items); SUD, substance-use disorder; SRAD, substance-related and addictive disorder.

For the original YFAS and the original mYFAS: Each symptom question has a specific threshold, as determined by Gearhardt et al. (3): ≥4 times/week for items 2 and 4, ≥2 times/week for items 5, 9, 12, 15, and 16, and “yes” for items 19 and 21.

For the YFAS 2.0 and mYFAS 2.0: Each symptom question has a specific threshold, as determined by Gearhardt et al. (13): Once a month for items 9, 10, 19, 27, 33, and 35; two to three times a month for items 8, 18, 20, 21, 34; once a week for items 3, 11, 13, 14, 22, 28, and 29; two to three times a week for items 5, 12, 16, 17, 23, 24, 26, 30, 31, and 32; four to six times a week for items 1, 2, 4, 6, 7, 15, and 25.

Both the YFAS 2.0 and the mYFAS 2.0 have been suggested to be unidimensional, and to have good internal consistency and convergent validity [see for the full YFAS 2.0 and mYFAS 2.0 English versions (13, 14); see for the full YFAS 2.0 other language versions, Arabic (15), Brazilian/Portugese (16), French (17), German (18), Italian (19), and Spanish (20); see for the mYFAS 2.0 other language versions, Italian (21), Portuguese/Brazilian (22)].

Several studies also demonstrated the close association between FA, as assessed by the YFAS 2.0, and psychiatric or psychological factors usually associated with SRAD [see Burrows et al. (23) and Penzenstadler et al. (24) for recent reviews]: impulsivity (25), especially attentional and motor impulsivity (26), higher levels of psychopathology (27–29), poorer emotion regulation skills (30), emotional eating (31), and psychiatric disorders such as mood and anxiety disorders (30, 32), eating disorders (33–35), eating disorder severity (30), and higher suicidality (32) and non-suicidal self-injury (33).

Yet, to our knowledge, whereas the psychometric properties of the YFAS 2.0 have been tested in clinical and non-clinical samples, those of the mYFAS 2.0 were examined exclusively in non-clinical populations (14, 21, 22, 36). Moreover, there is a lack of comparison of the psychometric properties of the mYFAS 2.0 versus full YFAS 2.0 in clinical populations. Finally, a validation of the mYFAS 2.0 for French-speaking populations is also needed.

This study’s primary objective was to assess the factor structure, internal consistency, and convergent validity of the French-speaking mYFAS 2.0 in a non-clinical population (study 1) and in patients with obesity (study 2). We also aimed to compare the FA prevalence, number of FA criteria, and types of FA criteria endorsed with the full YFAS 2.0 and the mYFAS 2.0 in each of these two populations. In the non-clinical population (study 1), we expected to confirm the unidimensionality of the mYFAS 2.0 and to demonstrate its good internal consistency and its good convergent validity, not only with the full YFAS 2.0 but also with measures of eating-related behaviors. We also hypothesized that the mYFAS 2.0 would perform similarly than the full YFAS 2.0 in terms of FA prevalence and number of FA symptoms detection. In the clinical population (study 2), we expected the mYFAS 2.0 to be unidimensional to have a good internal consistency and a good convergent validity not only with the full YFAS 2.0 but also with measures of eating-related behaviors and depression. As the mYFAS 2.0 was initially designed for large epidemiological studies in non-clinical samples, but not clinical samples, we aimed to determine if, in our clinical population, the mYFAS 2.0 and the full YFAS 2.0 would perform equally in terms of both FA prevalence and number of FA symptoms detection. We assumed that the full YFAS 2.0 would perform better than the mYFAS 2.0 in patients with obesity, but not in the non-clinical sample.

## Materials and Methods

### Study 1: mYFAS 2.0 Properties Among Non-Clinical Individuals

#### Participants and Procedures

We recruited a non-clinical sample of 250 participants from the community (non-underweight, no-obese persons that did not screened positive for any eating disorders) that stems from a larger sample of 330 persons that has been described previously (17). Participants were told that the study investigated eating behavior, they engaged freely in the study, and there was no financial compensation. This larger sample was recruited at the University of Tours between May 2014 and May 2015 using a web-based questionnaire that was created using Sphinx software [Sphinx Plus 2 version 5.1.0.4; (37)]. Out of these 330 initial participants, we excluded individuals that screened positive for anorexia nervosa (based on the eating disorder diagnostic scale; n = 5), bulimia nervosa (based on the questionnaire on eating and weight patterns-revised; n = 10), and binge eating disorder (based on the questionnaire on eating and weight patterns-revised; n = 14), as well as individuals who had either a body mass index (BMI) < 18.5 kg/m^2^ (underweight; n = 32) or a BMI equal to or greater than 30 kg/m2 (obesity; n = 28); 7 individuals had both a positive screening for an eating disorder and a BMI < 18.5 kg/m^2^; 2 persons had both a positive screening for an eating disorder and a BMI > 30 kg/m^2^. For more details about the cutoffs used to screen for eating disorders, see (17). **Table 2** presents the descriptive statistics of our final non-clinical sample (n = 250, including 50% of students and 50% of their family members). **Table 3** additionally reports the prevalence, mean number of FA symptoms, and type of FA criteria endorsed in this non-clinical sample.

**Table 2:** Descriptive statistics of the non-clinical (study 1) and clinical (study 2) samples.

	Study 1:Non-clinical population (*n = 250*)	Study 2: Patients with obesity*(n = 345)*
*Sociodemographic characteristics*		
Age, mean years and SD	28.4 ± 11.3	43.4 ± 10.3
Gender, % (N) of female	80% (200)	75.7% (261)
Marital status, % (N) of patients married or in a relationship	37.2% (93)	48.1% (166)
*Weight-related variables*		
Current BMI, mean kg/m^2^ and SD	22.8 ± 2.7	44.5 ± 7.0
Previous maximal BMI, mean kg/m^2^ and SD	24.2 ± 3.2	47.2 ± 7.2
*Binge eating*		
Mean BES score and SD	8.2 ± 7.0	11.5 ± 7.5
Persons at risk for binge eating disorder (BES score ≥ 18), % (N)	12.8% (32)	19.1% (66)
*Depression, mean BDI score and SD*	–	7.3 ± 6.1
*Eating behavior characteristics (TFEQ-R18)*		
Cognitive restraint, mean score and SD	12.0 ± 4.1	
Uncontrolled eating, mean score and SD	18.2 ± 5.1	–
Emotional eating, mean score and SD	6.5 ± 2.7	

BDI, 13-item Beck depression inventory; BES, Binge Eating Scale; BMI, Body Mass Index; FA, food addiction; TFEQ-R18, revised 18-item version of Three-Factor Eating Questionnaire.

**Table 3:** Non-clinical population (study 1): Comparison of the results obtained with the mYFAS 2.0 and the YFAS 2.0 (n = 250).

	mYFAS 2.0	YFAS 2.0	Comparison between mYFAS 2.0 and YFAS 2.0(p-value)
*FA prevalence, % (N)*	6.4% (16)	7.6% (19)	.25
*FA symptoms, % (N)*			.017
*No FA symptom*	57.6% (144)	70.4% (176)	
*One FA symptom*	23.6% (59)	17.2% (43)	
*>1 FA symptom*	18.8% (47)	12.4% (31)	
*FA severity, % (N)*			
No FA	93.6% (234)	92.4% (231)	–
Mild	2.8% (7)	2.8% (7)	–
Moderate	2.4% (6)	2.4% (6)	–
Severe	1.2% (3)	2.4% (6)	–
*FA prevalence by criteria, % (N)*			
Consumed more than planned	7.6% (19)	20.8% (52)	.001
Unable to cut down (=persistent desire or unsuccessful efforts to cut down or control consumption of certain foods)	7.6% (19)	10.8% (27)	.008
Great deal of time spent	10.0% (25)	12.0% (30)	.063
Important activities given up	3.2% (8)	6.4% (16)	.008
Use despite physical/emotional consequences	5.6% (14)	8.0% (20)	.03
Tolerance	4% (10)	5.2% (13)	.25
Withdrawal	5.6% (14)	8.0% (20)	.03
Use despite social/interpersonal consequences	7.2% (18)	8.8% (22)	.13
Failure in role obligations	0.8% (2)	1.6% (4)	.50
Use in physically hazardous situations	2.4% (6)	7.6% (19)	.001
Craving	6% (15)	7.6% (19)	.13
Impairment or distress	9.2% (23)	9.2% (23)	1

FA, food addiction; mYFAS 2.0, modified version of the Yale Food Addiction Scale 2.0 (based on the DSM-5 criteria for substance-related and addictive disorders; 13 items); YFAS 2.0, Yale Food Addiction Scale 2.0 (based on the DSM-5 criteria for substance-related and addictive disorders; 35 items).

#### Ethical Considerations

For this study, we obtained the approval of a local institutional review board (Tours). We performed all procedures in accordance with the ethical standards described in the 1964 Declaration of Helsinki and its later amendments, as revised in 2013. All participants provided written informed consent prior to their inclusion in the study.

#### Measurements

We assessed socio-demographic characteristics (i.e., age, gender, and marital status). Self-reported height, current weight, and maximal weight were used to calculate current BMI and previous maximal BMI.

##### Yale Food Addiction Scale Version 2.0 and Modified Yale Food Addiction Scale 2.0

We used the French version of the Yale Food Addiction scale 2.0 [(17); original version: (13)]. It includes 35 items with two to five questions assessing each of these 11 DSM-5 SRAD criteria in addition to clinically significant impairment or distress, as applied to foods high in fat and/or refined carbohydrates [for more details about the items belonging to each criterion, see Gearhardt et al. (13)]. All questions on the YFAS 2.0 are continuous and have eight frequency response options that range from “Never” (= 0) to “Every Day” (= 7). To reflect diagnostic thresholds, a cutoff for each question was applied to allow for determination of a diagnosis and severity level [see Gearhardt et al., (13) for more details]. For the YFAS 2.0 FA “diagnosis” scoring option, both the symptom count score and the clinical significance criterion are used. The determination of a FA “diagnosis” and its severity level is as follows: no FA if one or fewer symptoms or they do not meet criteria for clinical significance; mild FA if two or three symptoms and clinical significance, moderate FA if four or five symptoms and clinical significance, and severe FA if six or more symptoms and clinical significance.

Akin to the development of the mYFAS (12) from the original YFAS and the mYFAS 2.0 (14) from the YFAS 2.0, the French-speaking mYFAS 2.0 was derived from the French full YFAS 2.0. Items selection for the French-speaking mYFAS 2.0 was based on the same items than those proposed by Schulte and Gearhardt (14) for their original mYFAS 2.0.

##### Binge Eating Scale

The Binge Eating Scale [BES; (38)] encompasses 16 items designed to assess the severity of binge eating using behavioral, affective, and cognitive symptoms. We considered binge eating as a continuous variable and as a categorical variable (significant binge eating if BES score ≥ 18). We used the validated French version (39). In our sample, Cronbach’s α was .88.

##### The Revised 18-Item Version of the Three Factor Eating Questionnaire

We used the revised 18-item version of the Three Factor Eating Questionnaire (TFEQ-R18) [original version: (40); French version: (41)] to assess uncontrolled eating (tendency to eat more than usual due to a loss of control over intake accompanied by subjective feelings of hunger), emotional eating (the propensity to eat more in response to emotional triggers), and cognitive restraint (conscious restriction of food intake in order to control body weight or to promote weight loss). We followed the scoring options for the French version described by de Lauzon et al. (41). In our sample, Cronbach’s α were, respectively, .83 for cognitive restraint, .80 for uncontrolled eating, and .86 for emotional eating.

#### Statistical Analyses

All analyses were conducted using the *R* statistical package version 3.5.0 (42) with the psych (43) package, except for confirmatory factor analyses that were conducted using the Mplus software (44). Statistical analyses included descriptive statistics, examination of the psychometric properties of the scale (factor structure, internal consistency, convergent validity) and comparison of the results obtained with the short or full versions of the scale (i.e., French-speaking mYFAS 2.0 vs French-speaking YFAS 2.0).

The unidimensionality of the mYFAS 2.0 has been previously demonstrated using an exploratory factor analysis in non-clinical populations (14). To test whether this applies to the French-speaking mYFAS 2.0, we used a first-order confirmatory factor analysis (CFA) based on the 11 diagnostic criteria (each item being considered as a binary variable, like in the mYFAS 2.0 English validation study), with the use of tetrachoric correlations to take into account the dichotomous nature of the data. CFA provides fit indices that assess how the theoretical model fits to the data. We followed the recommendations of Kline (45) and Bentler (46) and used the following indices and commonly reported cutoffs: Comparative fit index (CFI) above 0.90, root mean square error of approximation (RMSEA) under 0.05, and chi-square and adjusted chi-square (i.e., chi-square divided by the degrees of freedom) under 3. For this fit indices, we used a weighted least square means and variance adjusted estimation, which is a robust estimator that does not assume normally distributed variables and provides the best option for modelling categorical data (47).

To assess the internal consistency of the scale, we used Kuder-Richardson alpha (KR-20) and McDonald’s omega. McDonald’s omega is an estimate of the general factor saturation of a test (i.e., in this case, factor analysis); it is an estimate of the scale reliability that has been shown to be a more sensible index of internal consistency—both in relation to alpha and also when compared to other alternatives (48). We used Kayser-Meyer-Olkin (KMO) statistics to assess the sampling adequacy. In line with Falissard et al. recommendation (49), we also evaluated the scale reliability by calculating inter-item correlations and correlations between each individual item and the domain scores omitting the item [correlation values above .20 between an item and its domain score were considered satisfactory; (50)].

After inspection of the distribution of the mYFAS 2.0 scores (skewness = 3.01 ± .15 and kurtosis = 10.35 ± .31) and to assess its convergent validity, we examined the associations with the number of FA symptoms (full French-speaking YFAS 2.0), current BMI, previous maximal BMI, binge eating (BES score), emotional eating, uncontrolled eating, and cognitive restraint (TFEQ-R18 sub-scores) using Spearman’s correlation coefficients. We determined the association between FA diagnosis and the risk of presenting a binge eating disorder (as defined by a BES score ≥ 18) using chi-squared tests (Fisher tests), as well as between FA type (no FA and mild FA vs. moderate and severe FA) and the risk of presenting a binge eating disorder (as defined by a BES score ≥ 18) using chi-squared tests (Fisher tests).

To compare the results obtained with the full and shortened version of the scale, first, in terms of FA “diagnosis” prevalence and prevalence for each of the 11 FA criteria, we used McNemar’s tests; second, for the number of FA symptoms endorsed, we used a Wilcoxon signed-rank test.

### Study 2: mYFAS 2.0 Properties Among Patients With Obesity

#### Participants and Procedures

This cross-sectional study included 345 patients that were candidates for bariatric surgery in three wards specialized in severe obesity (Québec, Canada n = 35, 10.1%; Reims, France n = 121, 35.1%; Tours, France n = 189; 54.8%). The participants were told that the study would enable a better understanding of the difficulties encountered by bariatric surgery candidates, and there was no financial compensation. Although the study was proposed before the approval process for the surgery, they were also told that their results would not impact their treatment and the selection process. The assessment was conducted during the preoperative assessment. **Table 2** presents the descriptive statistics of the study population sample. **Table 4** additionally reports the prevalence, mean number of FA symptoms and type of FA criteria endorsed in this clinical sample. There was no significant difference in terms of FA prevalence between the three centers (Québec=14.3%; Reims=20.7%; Tours=21.7%; p=.61).

**Table 4:** Factor loadings of the modified Yale Food Addiction Scale 2.0 (mYFAS 2.0) in the non-clinical sample and in patients with obesity (confirmatory factor analyses with tetrachoric correlations based on dichotomous data).

	Corresponding mYFAS 2.0 dichotomous item	Study 1 (non-clinical population): factor loadings of the one-factor solution	Study 2 (patients with obesity): factor loadings of the one-factor solution
*Food consumed in larger quantities or over a longer period than intended*	#3	.56	.56
*Persistent desire or unsuccessful efforts to cut down or control consumption of certain foods*	#32	.67	.67
*Considerable time spent to obtain, consume, or recover from effects of food*	#5	.61	.61
*Giving up important social, occupational, or recreational activities because of food consumption*	#10	.45	.45
*Continuing to eat certain foods despite physical or psychological problems*	#22	.83	. 83
*Tolerance*	#24	.49	.49
*Withdrawal*	#13	.64	.64
*Continued use despite social or interpersonal problems*	#35	.66	.66
*Failure to fulfill major role obligations*	#19	.64	.48
*Eating certain foods in physically hazardous situations*	#33	.48	.48
*Craving*	#29	.80	.80
*Impairment or distress*	#16 and #17	Not included	Not included

mYFAS 2.0, modified version of the Yale Food Addiction Scale 2.0 (based on the DSM-5 criteria for substance-related and addictive disorders; 13 items); YFAS 2.0, Yale Food Addiction Scale 2.0 (based on the DSM-5 criteria for substance-related and addictive disorders; 35 items).

#### Ethical Considerations

This study was approved by the Institutional Review Board of the University Hospital of Tours, France (Ethics Committee in Human Research, IRB number: 2018-057), as well as the Québec Heart and Lung Institute Research Center research ethics committee, Québec City, Canada (2016-2569, 21237), and the Institutional Review Board of the University Hospital of Reims, France (Ethics Committee in Human Research, IRB number: 2016-12). The data were collected in line with the recommendations regarding use of personal data, especially with the approval of the French CNIL (Commission Nationale de l’Informatique et des Libertés). All participants provided written informed consent after the procedure was explained and prior to their inclusion in the study.

#### Measurements

We collected socio-demographic characteristics (i.e., age, gender, marital status), current BMI, previous maximal BMI from medical records. In addition to the full YFAS 2.0, the mYFAS 2.0 and the Binge Eating Scale (see the Study 1 Method section), the patients completed the 13-item Beck Depression Inventory [original version: (51); French version: (52)]. In this sample, Cronbach’s alpha for the BES and BDI were respectively .83 and .86.

#### Statistical Analyses

Analyses were conducted using the *R* statistical package version 3.5.0 (42) with the psych package (43), except for confirmatory factor analyses that were conducted using the Mplus software (44). Statistical analyses included descriptive statistics, examination of the mYFAS 2.0 factor structure, internal consistency and convergent validity, as well as a comparison of the FA prevalence, number of FA symptoms and prevalence for each diagnostic criterion obtained with the mYFAS 2.0 and full YFAS 2.0.

For the factor structure of the French-speaking mYFAS 2.0, which was found to be unidimensional in non-clinical populations, we conducted a first-order confirmatory factor analysis for dichotomous data [i.e., each of the 11 FA diagnostic criteria; see Gearhardt et al. (13)] based on tetrachoric correlation coefficients and using an oblique rotation (same procedure as in Study 1). For internal consistency and sampling adequacy, we used the same procedure than in Study 1.

After inspection of the distribution of the mYFAS 2.0 scores (skewness=1.59 ± .13 and kurtosis=2.24 ± .26), and to assess its convergent validity, we tested its association with the number of full YFAS 2.0 FA symptoms, the current BMI and previous maximal BMI, the severity of binge eating and depressive symptoms using Spearman’s rank correlation coefficients. We determined the association between FA diagnosis and the risk of presenting a binge eating disorder (as defined by a BES score ≥ 18) using chi-squared tests (Fisher tests), as well as between FA type (no FA and mild FA vs. moderate and severe FA) and the risk of presenting a binge eating disorder (as defined by a BES score ≥ 18) using chi-squared tests (Fisher tests).

To compare the results obtained with the full and shortened version in terms of FA “diagnosis” prevalence and prevalence for each of the 11 FA criteria, we used McNemar’s tests; for the number of FA symptoms endorsed, we used a Wilcoxon signed-rank test.

## Results

### Study 1: mYFAS 2.0 Properties Among Non-Clinical Individuals

#### mYFAS 2.0 Factor Structure and Internal Consistency

The CFA yielded the following goodness of fit indices (one-factor structure): χ2 = 43.68, df (degrees of freedom) = 44, χ^2^/df = .99, p = .49; CFI = 1.00, and RMSEA = .00 90% CI [.001–.042]. All factor loadings were greater than .50 (**Table 4**). This one-factor model explained 47.6% of the total variance. The internal consistency (KR-20 = .78, Mc Donald’s omega = .83) and sampling adequacy (KMO = .79) were satisfactory. The results mainly supported the mYFAS 2.0 has a one-factor structure in this non-clinical population. Inter-item correlations were all above .38, and correlations between each individual item and the domain scores omitting the item were all above .26 and thus considered satisfactory.

#### Convergent Validity of the mYFAS 2.0

As presented in **Table 5**, the mYFAS 2.0 symptom score was positively and significantly correlated with the YFAS 2.0 symptom score, the current and previous maximal BMI, the severity of binge eating, cognitive restraint, uncontrolled eating, and emotional eating, with coefficient values close to those obtained with the full YFAS 2.0 symptom score. FA “diagnosis” was significantly associated with the risk of binge eating disorder (p <.001), with 9 participants out of the 16 with a FA ‘diagnosis’ (i.e., 56.3%) who endorsed a BES score ≥ 18 (**Table 6**). Participants with a severe or moderate FA were more likely to be at risk for binge eating disorder than persons with mild FA or no FA (p <.001). However, in the subgroup of patients at risk for binge eating disorder, 71.9% had no FA, while 28.1% had FA (**Table 6**).

**Table 5:** Non-clinical population (study 1): Correlation matrix (Spearman’s rank correlation coefficients).

	1	2	3	4	5	6	7	8
1. mYFAS 2.0 score	–							
2. YFAS 2.0 score	.83^***^	–						
3. Current BMI	.17^**^	.16^*^	–					
4. Previous maximal BMI	.16^*^	.15^*^	.85^***^	–				
5. Binge eating (BES)	.42^***^	.47^***^	.21^***^	23^***^	–			
6. Cognitive restraint (TFEQ-R18)	.24^***^	.22^***^	.22^***^	.32^***^	.48^***^	–		
7. Uncontrolled eating (TFEQ-R18)	.30^***^	.36^***^	.06	.04	.68^***^	.24^***^	–	
8. Emotional eating (TFEQ-R18)	.31^***^	.30^***^	.12	.17^**^	.64^***^	.41^***^	.52^***^	–

BES, Binge Eating Scale; BMI, body mass idex; TFEQ-R18, revised 18-item version of Three-Factor Eating Questionnaire; YFAS 2.0, Yale Food Addiction Scale version 2.0 (based on the DSM-5 criteria for substance-related and addictive disorders; 35 items); mYFAS 2.0, modified Yale Food Addiction Scale 2.0 (based on the DSM-5 criteria for substance-related and addictive disorders; 13 items). ^*^ p <.05; ^***^p <.01; ^***^p <.001.

**Table 6:** Association between FA severity and risk for BED in the non-clinical sample and in patients with obesity.

Non clinical population (study 1; n = 250)		
FA status	BED status	
	At risk for BED (BES score ≥ 18)	Not at risk for BED (BES score < 18)
*FA severity (mYFAS 2.0), % (N)*		
No FA	71.9% (23)	96.8% (211)
Mild	12.5% (4)	1.4% (3)
Moderate	6.3% (2)	1.8% (4)
Severe	9.4% (3)	0% (0)
*FA severity (full YFAS 2.0), % (N)*		
No FA	68.8% (22)	95.9% (209)
Mild	6.3% (2)	2.3% (5)
Moderate	12.5% (4)	0.9% (2)
Severe	12.5% (4)	0.9% (2)
**Patients with obesity and candidates for bariatric surgery (study 2; n = 345)**		
*FA severity (mYFAS 2.0), % (N)*		
No FA	50% (33)	86.4% (241)
Mild	15.2% (10)	8.2% (23)
Moderate	12.1% (8)	4.3% (12)
Severe	22.7% (15)	1.1% (3)
*FA severity (full YFAS 2.0), % (N)*		
No FA	36.4% (24)	82.8% (231)
Mild	15.2% (10)	6.5% (18)
Moderate	16.7% (11)	5.4% (15)
Severe	31.8% (21)	5.4% (15)

BED, binge eating disorder; FA, food addiction; mYFAS 2.0, modified version of the Yale Food Addiction Scale 2.0 (based on the DSM-5 criteria for substance-related and addictive disorders; 13 items); YFAS 2.0, Yale Food Addiction Scale 2.0 (based on the DSM-5 criteria for substance-related and addictive disorders; 35 items).

#### Comparison Between the mYFAS 2.0 and the Full YFAS 2.0

**Table 3** presents the FA prevalence, number of FA symptoms and prevalence for each diagnostic criterion obtained with the full YFAS 2.0 and mYFAS 2.0. The FA prevalence did not differ significantly between the full YFAS 2.0 and the mYFAS 2.0 (7.6% vs. 6.4%; p = .25), but the number of FA symptoms was significantly higher for the full YFAS 2.0 than the mYFAS 2.0 (1.0 ± 1.7 vs. 0.6 ± 1.3; Z = -7.51, N-Ties = 67, p <.001). The mYFAS 2.0 and full YFAS 2.0 prevalence for each diagnostic criterion did not differ for the “Great deal of time spent”, “Tolerance”, “Use despite social/interpersonal consequences”, “Failure in role obligation”, and “Craving” criteria, but they were significantly higher for the full YFAS 2.0 for the six remaining FA criteria (**Table 3**).

### Study 2: mYFAS 2.0 Properties Among Patients With Obesity

#### mYFAS 2.0 Factor Structure and Internal Consistency

The CFA yielded the following goodness of fit indices (one-factor structure): χ^2 ^= 46.53, df (degrees of freedom) = 40, χ^2^/df = 1.16, p = .22; CFI = .989, and RMSEA = .022 90% CI [.001–.045]. All factor loadings were greater than .45 (**Table 4**). This one-factor model explained 31.2% of the total variance, while a two-factor structure only slightly improved the percentage of total variance explained (13.5%) with no better fit indices. The internal consistency (KR-20 = .73, Mc Donald’s omega = .77) and sampling adequacy (KMO = .76) were satisfactory. Thus, the results mainly supported the mYFAS 2.0 has a one-factor structure in this clinical population. Inter-item correlations were all above .43, and correlations between each individual item and the domain scores omitting the item were all above .27 and thus considered satisfactory.

#### mYFAS 2.0 Convergent Validity

As presented in **Table 7**, the mYFAS 2.0 symptom score was positively and significantly correlated with the full YFAS 2.0 symptom score, the severity of binge eating, and depression, with coefficient values close to those obtained with the full YFAS 2.0. The mYFAS 2.0 symptom score was not correlated neither with current BMI nor with previous maximal BMI, while the full YFAS 2.0 score was correlated with current BMI. FA “diagnosis” was significantly associated with the risk of binge eating disorder (p <.001), with 38 patients out of the 71 with a FA “diagnosis” (i.e., 46.5%) who endorsed a BES score ≥ 18 (**Table 6**). Patients with a severe or moderate FA were more likely to be at risk for binge eating disorder than patients with mild FA or no FA (p <.001). However, in the subgroup of patients at risk for binge eating disorder, 50% had no FA, while 50% had FA (**Table 6**).

**Table 7:** Patients with obesity (study 2): Correlation matrix (Spearman’s rank correlation coefficients).

	1	2	3	4	5	6
1. mYFAS 2.0 score	–					
2. YFAS 2.0 score	.81^***^	–				
3. Current BMI	.07	.11^*^	–			
4. Previous maximal BMI	.03	.04	.82^***^	–		
5. Binge eating (BES)	.47^***^	.45^***^	-.04	-.03	–	
6. Depression (BDI)	.34^***^	.35^***^	.11^*^	.11^*^	.52^***^	–

BDI, 13-item beck depression inventory; BES, Binge Eating Scale; BMI, body mass index; YFAS 2.0, Yale Food Addiction Scale version 2.0 (based on the DSM-5 criteria for substance-related and addictive disorders; 35 items); mYFAS 2.0, modified Yale Food Addiction Scale 2.0 (based on the DSM-5 criteria for substance-related and addictive disorders; 13 items). ^*^p <.05; ^***^p <.001.

#### Comparison Between the mYFAS 2.0 and the Full YFAS 2.0

There was a significant difference between the full YFAS 2.0 and the mYFAS 2.0 in terms of FA prevalence (26.1% vs. 20.6%; p = .001) and number of FA symptoms (2.4 ± 2.6 vs. 1.6 ± 2.0; Z = -11.43, N-Ties = 167, p <.001) (see **Table 8**). The mYFAS 2.0 and full YFAS 2.0 prevalence for each diagnostic criterion were not significantly different for the “Craving” and “Failure in role obligations” criteria, but they were significantly higher for the full YFAS 2.0 for the remaining nine FA criteria (**Table 8**).

**Table 8:** Patients with obesity (study 2): Comparison of the results obtained with the mYFAS 2.0 and the full YFAS 2.0 (n = 345).

	mYFAS 2.0	YFAS 2.0	Comparison between mYFAS 2.0 and YFAS 2.0(p-value)
*FA prevalence, % (N)*	20.6% (n = 71)	26.1% (n = 90)	.001
*FA symptoms, mean score and SD*	1.6 ± 2.0	2.4 ± 2.6	.001
*FA severity, % (N)*			
Mild	9.6% (n = 33)	8.1% (n = 28)	–
Moderate	5.8% (n = 20)	7.5% (n = 26)	–
Severe	5.2% (n = 18)	10.4% (n = 36)	–
*FA prevalence by criteria, % (N)*			
Consumed more than planned	13.0% (n = 45)	27.2% (n = 94)	.001
Unable to cut down (= persistent desire or unsuccessful efforts to cut down or control consumption of certain foods)	19.1% (n = 66)	29.6% (n = 102)	.001
Great deal of time spent	18.6% (n = 64)	21.2% (n = 73)	.004
Important activities given up	7.5% (n = 26)	17.7% (n = 61)	.001
Use despite physical/emotional consequences	14.2% (n = 49)	24.6% (n = 85)	.001
Tolerance	18.8% (n = 65)	21.7% (n = 75)	.002
Withdrawal	13.0% (n = 45)	23.8% (n = 82)	.001
Use despite social/interpersonal consequences	21.4% (n = 74)	24.6% (n = 85)	.001
Failure in role obligations	16.8% (n = 58)	18.3% (n = 63)	.063
Use in physically hazardous situations	4.3% (n = 15)	24.1% (n = 83)	.001
Craving	9.9% (n = 34)	11.3% (n = 39)	.063
Impairment or distress	29.6% (n = 102)	29.6% (n = 102)	1

FA, food addiction; mYFAS 2.0, modified version of the Yale Food Addiction Scale 2.0 (based on the DSM-5 criteria for substance-related and addictive disorders; 13 items); YFAS 2.0, Yale Food Addiction Scale 2.0 (based on the DSM-5 criteria for substance-related and addictive disorders; 35 items).

## Discussion

These studies aimed to assess the psychometric properties of the mYFAS 2.0 and to compare the results obtained with the mYFAS 2.0 and the full YFAS 2.0 in both a non-clinical population (study 1) and in patients with obesity and candidates for bariatric surgery (study 2). One of the main implications of our findings is that our results were in line with the one-factor structure of the mYFAS 2.0 and the YFAS 2.0 and that the mYFAS 2.0 and the YFAS 2.0 should be differentially interpreted and used depending on the population studied (non-clinical or clinical population) and the type of measurement used (FA prevalence or FA symptom count). First, in non-clinical populations, our study suggests that the mYFAS 2.0 can be used as a reliable proxy for the full YFAS 2.0 to assess FA prevalence. However, relative to the full YFAS 2.0, it tends to underestimate the number of FA symptoms. Therefore, we propose that the mYFAS 2.0 should be used as a descriptive or screening tool in non-clinical samples. Second, in treatment-seeking patients with obesity, we demonstrated that the mYFAS 2.0 performed differently than the full YFAS 2.0 in terms of both FA prevalence and number of FA symptoms. This suggests that while the use of the mYFAS 2.0 can be recommended in a non-clinical population to assess FA prevalence or for screening addictive-like eating, caution should be exercised when using it among clinical groups, where the full YFAS 2.0 should be preferred to limit the risk of false negatives.

Our study also makes substantial contribution to the evolving field of research on addictive-like eating. On the one hand, it provides a validation of a French-speaking version of the mYFAS 2.0, which we believe is particularly suited for use in large epidemiological studies where participant burden has to be considered. On the other hand, it adds to the literature on whether FA should be conceptualized as a subtype of binge eating disorder.

We observed that the mYFAS 2.0 had psychometric properties similar to those of the full YFAS 2.0: unidimensional, with an acceptable internal consistency and good convergent validity. Our study confirmed the one-factor structure of the mYFAS 2.0 previously observed in non-clinical populations (14, 21, 22). We additionally demonstrated that this one-factor structure was also suitable in a sample of patients with obesity who are candidates for bariatric surgery. Convergent validity indices were also in line with previous studies using the YFAS 2.0 and mYFAS 2.0 symptom scores, with an association with binge eating and emotional eating severity, as well as a positive but moderate association with current and previous maximum BMI (this latter being observed only in the non-clinical population) (13, 14, 20–22). We also replicated the reported positive association between the YFAS 2.0 symptom score and the level of psychological distress in bariatric surgery candidates (30).

Among the non-clinical sample, mYFAS 2.0 FA ‘diagnosis’ prevalence was 6.4%, which is lower than what has been previously reported in US non-clinical populations (ranging from 13.1 to 15% according to Schulte and Gearhardt (11, 14), similar to the results from an Italian sample [5.7%; Imperatori et al., (21)] but slightly higher than those from a Brazilian sample [4.3%; (22)]. The fact we observed a lower FA diagnosis prevalence than the one reported in studies with US samples might be due to our exclusion of participants that screened positive for disordered eating behaviors (as assessed by the questionnaire on eating and weight patterns-revised and the eating disorder diagnostic scale) and of people with a BMI under 18.5 or over 30. This suggestion is in line with the results from Granero et al. (20) who reported a lower FA prevalence in individuals without an eating disorder than in those with an eating disorder; it is also consistent with Meule’s recent hypothesis that there might be a cubic relationship between FA and BMI (53). An alternative explanation might be the cross-cultural differences in terms of eating patterns, relationship to food, and/or availability/accessibility/type of food items (54), which might subsequently modulate the risk of disordered eating behaviors/FA.

As expected, among the clinical sample, the prevalence of people endorsing a FA ‘diagnosis’ using the mYFAS 2.0 (20.6%) was greater than in our non-clinical sample (6.1%). Yet, as this is the first study to assess the prevalence of a FA ‘diagnosis’ using the mYFAS 2.0 in such population, this prevents any further comparison with the literature. However, this is consistent with the results obtained in previous studies using the DSM-IV-TR based mYFAS and YFAS (24, 55). Future studies should be conducted in patients with obesity outside the field of bariatric surgery to confirm the psychometric properties of the mYFAS 2.0 in this broader population.

Regarding the YFAS 2.0, previous studies conducted in people seeking treatment for obesity found an FA prevalence that ranged from 6.7 to 47.4% (18, 30, 56, 57). These differences in FA prevalence might be explained by differences in terms of age (i.e., rates are higher in middle adulthood, followed by young adulthood, and lowest in elder adults), gender (i.e., greater among females), and sample type (greater among patients with an eating disorder) (23, 24). Of note, with regard more specifically to bariatric surgery patients, difference in prevalences may also be due to when and how the self-report is completed (how the study is presented to participants; study done before or after the approval process for the surgery; whether the assessment is part of the approval process or not): when the assessment is part of the presurgical evaluation interview, this may lead participants to selectively underreport some of their symptoms in order to present themselves as psychologically healthy candidates (58), or possibly to report less psychological symptoms because some of them may perceive that they are finally accessing a reliable solution to their lifelong problem. Our 26.1% prevalence is similar to the 27.3% prevalence observed by Müller et al. (30) and the 26.4% prevalence reported by Guerrero Pérez et al. (57) in a comparable population in terms of age, gender-ratio, BMI, and type of sample (bariatric surgery candidates). We may thus assume that our findings could be extrapolated to the population of bariatric surgery candidates. Nevertheless, in our clinical sample the majority (52.2%) of the FA diagnosis were of mild severity, whereas in the Meule et al. (18) and the Müller et al. (30) studies, the majority of the FA diagnosis were classified in the severe category (44 and 54.3%, respectively). FA severity is not systematically reported (e.g., not reported in the Guerrero Pérez et al. (57) study), and it is rarely considered as a potential critical factor. As some factors may be differentially associated to FA depending on its severity itself [(e.g., see Carter et al. (34)], we too recommend to report and analyze FA severity in addition to FA prevalence and number of FA symptoms in future studies to enhance the comparability of measurement results.

Another interesting point of our results pertains to the ongoing debate on the assessment of ‘FA’ and ‘binge eating’ as either similar or separate constructs/measures (59, 60). Using both the short and full YFAS 2.0, we found that a larger number of FA symptoms was related to more severe binge eating, but with an association of medium magnitude for both samples (i.e. rho <.50) on the one hand, and only a partial overlap between the two measures (half of the participants who endorsed a FA diagnosis had significant binge eating) on the other hand. In addition, with respect to the participants who had significant binge eating, less than one third of them (28.1%) in the non-clinical sample and 50% of them in the clinical sample endorsed a FA diagnosis. Of note, previous studies found that FA severity was associated with a more severe binge eating symptomatology (34). We confirmed here this result in both a non-clinical population and in patients seeking a surgical treatment for their obesity. Although we assessed binge eating symptoms, and not binge eating disorder (as a psychiatric categorical diagnosis, which has a different definition), our results are in line with previous research and also support the view of two different clinical entities (61, 62). To support this hypothesis, the next step will be to confirm the association of either FA or binge eating/binge eating disorder with different outcomes. In this context, it would be appropriate to consider FA severity as a potential predictor for other outcome variables, notably eating disorder diagnosis. If confirmed, then FA may then be conceptualized either as a standalone disorder, or as a transdiagnostic construct that could be an additional specifier among individuals with binge eating disorder (i.e., patients with binge eating disorder could benefit from different therapeutic strategies depending on their FA status) (59). This is also in line with the idea that future research could be more fruitful if the focus was on identifying overlapping and distinctive underlying mechanisms between FA and binge eating disorder rather than similarities and differences in clinical features (34, 63).

This study has a number of limitations. As we used no specific compensation to increase the number of potential participants, we cannot rule out the hypothesis that our samples may represent a particularly generous, pro-social subsets that may not be representative of a study population as a whole. Another limit pertains to the assessment of the FA concept: if the addictive model of food/eating was confirmed, future studies should determine whether the “FA” phenotype should be best understood as a substance-use disorder (13) or as a behavioral addiction (i.e., eating addiction rather than “FA”) (64, 65), and how it should be included in the international diagnostic classifications with regard to traditional eating disorders. Another limitation is the weight status that was self-reported in study 1, because self-reported weight tends to be overestimated in men and underestimated in women (66). Moreover, the specificity of our clinical population should be pointed (patients seeking treatment for their obesity and who were candidates for bariatric surgery): future studies should test the mYFAS 2.0 psychometric properties in patients with less severe obesity or with non-severe obesity before generalizing our results to all individuals with obesity (including non-bariatric populations), as well as in patients with eating disorders or higher levels of emotional eating or restrained eating; these studies with both the mYFAS 2.0 and the YFAS 2.0 would be useful to better understand the differences in terms of FA prevalence in the clinical population and to test whether the YFAS 2.0 has the same one-factor structure in these populations. Our study included 20% of men in study 1 and 24.3% in study 2. Although the gender-ratio observed in our clinical sample was comparable to those reported in similar studies with patients consulting for obesity (30, 57, 67), additional studies, with a more equilibrated gender-ratio are needed and could also investigate the measurement invariance across gender (68). We may assume that such studies could benefit from a systematic assessment and exclusion of participants with eating disorders, given that this could biases results of studies conducted in non-clinical populations if not addressed. Finally, the absence of alternative measures for the diagnosis or the quantification of FA precluded accurate analysis of convergent validity or the determination of cutoffs using a ROC curve; future studies could also test the convergent validity of the mYFAS 2.0 with other measures of addictive-like eating.

In conclusion, this study demonstrated that the mYFAS 2.0 had psychometric properties close to those of the full YFAS 2.0 in both a non-clinical sample and in treatment seeking patients with obesity: unidimensionality, acceptable to good internal consistency and good convergent validity. Although valid and reliable in patients with obesity, our results demonstrated that the use of the mYFAS 2.0 in this clinical population might lead to a significant underestimation of FA prevalence and number of FA symptoms when compared to the full YFAS 2.0. Use of the YFAS 2.0 and mYFAS 2.0 in future studies will enable a better delineation of the limits of the FA concept and its potential predictive validity over important outcomes measurements after treatment.

## Data Availability Statement

The datasets generated for this study are available on request to the corresponding author.

## Ethics Statement

The studies involving human participants were reviewed and approved by (1) Institutional Review Board of the University Hospital of Tours, France (Ethics Committee in Human Research, IRB number: 2018-057), (2) Québec Heart and Lung Institute Research Center research ethics committee, Québec City, Canada (2016-2569, 21237), and (3) Institutional Review Board of the University Hospital of Reims, France (Ethics Committee in Human Research, IRB number: 2016-12). The patients/participants provided their written informed consent to participate in this study.

## Author Contributions

Study design: PB, SB, RC, NB, FB, and CB. Analysis: PB. Writing the manuscript and critical comment to the drafts: PB, SB, AG, FG, AK, EB, AT, LB, AL, RH, RC, NB, and FB. All authors contributed to the article and approved the submitted version.

## Funding 

This research received no specific grant from any funding agency in the public, commercial, or not-for-profit sectors. We thank the University Hospital of Tours for paying the open access publication fees.

## Conflict of Interest

PB reports personal fees from Lundbeck, personal fees from Astra-Zeneca, and personal fees from D&A Pharma, outside the submitted work. NB reports personal fees from Lundbeck, personal fees from Astra-Zeneca, and personal fees from D&A Pharma, outside the submitted work. FB reports personal fees from Eutherapie and from Lundbeck, outside the submitted work. CB reports personal fees from Takeda outside the submitted work. AB and LB receive research funding from Johnson & Johnson Medical Companies and Medtronic for studies related to bariatric surgery.

The remaining authors declare that the research was conducted in the absence of any commercial or financial relationships that could be construed as a potential conflict of interest.

## References

[1] Ahmed SH Avena NM Berridge KC Gearhardt AN Guillem K “Food Addiction,” In: Pfaff DW, editor. Neuroscience in the 21st Century: From Basic to Clinical. New York, NY: Springer New York (2010). p. 2833–57. 10.1007/978-1-4614-1997-6_110

[2] Cocores JA Gold MS The Salted Food Addiction Hypothesis may explain overeating and the obesity epidemic. Med Hypotheses (2009) 73:892–9. 10.1016/j.mehy.2009.06.049 19643550

[3] Gearhardt AN Corbin WR Brownell KD Food addiction: an examination of the diagnostic criteria for dependence. J Addict Med (2009) 3:1–7. 10.1097/ADM.0b013e318193c993 21768996

[4] Volkow ND Wang GJ Fowler JS Tomasi D Baler R Food and drug reward: overlapping circuits in human obesity and addiction. Curr Top Behav Neurosci (2012) 11:1–24. 10.1007/7854_2011_169 22016109

[5] Meule A Back by Popular Demand: A Narrative Review on the History of Food Addiction Research. Yale J Biol Med (2015) 88:295–302. 26339213 PMC4553650

[6] Fletcher PC Kenny PJ Food addiction: a valid concept? Neuropsychopharmacol Off Publ Am Coll Neuropsychopharmacol (2018) 43:2506–13. 10.1038/s41386-018-0203-9 PMC6224546 30188514

[7] Cope EC Gould E New Evidence Linking Obesity and Food Addiction. Biol Psychiatry (2017) 81:734–6. 10.1016/j.biopsych.2017.02.1179 28391804

[8] Meule A Gearhardt AN Five years of the Yale Food Addiction Scale: Taking stock and moving forward. Curr Addict Rep (2014) 1:193–205. 10.1007/s40429-014-0021-z

[9] Gearhardt AN Corbin WR Brownell KD Preliminary validation of the Yale Food Addiction Scale. Appetite (2009) 52:430–6. 10.1016/j.appet.2008.12.003 19121351

[10] Gordon EL Ariel-Donges AH Bauman V Merlo LJ What Is the Evidence for “Food Addiction?” A Systematic Review. Nutrients (2018) 10:477. 10.3390/nu10040477 PMC5946262 29649120

[11] Schulte EM Smeal JK Lewis J Gearhardt AN Development of the highly processed food withdrawal scale. Appetite (2018) 131:148–54. 10.1016/j.appet.2018.09.013 30227182

[12] Flint AJ Gearhardt AN Corbin WR Brownell KD Field AE Rimm EB Food-addiction scale measurement in 2 cohorts of middle-aged and older women. Am J Clin Nutr (2014) 99:578–86. 10.3945/ajcn.113.068965 PMC3927691 24452236

[13] Gearhardt AN Corbin WR Brownell KD Development of the Yale Food Addiction Scale Version 2.0. Psychol Addict Behav (2016) 30:113–21. 10.1037/adb0000136 26866783

[14] Schulte EM Gearhardt AN Development of the Modified Yale Food Addiction Scale Version 2.0. Eur Eat Disord Rev (2017) 25:302–8. 10.1002/erv.2515 28370722

[15] Fawzi M Fawzi M Validation of an Arabic version of the Yale Food Addiction Scale 2.0. East Mediterr Health J (2018) 24:745–52. 10.26719/2018.24.8.745 30328605

[16] Nunes-Neto PR Köhler CA Schuch FB Solmi M Quevedo J Maes M Food addiction: Prevalence, psychopathological correlates and associations with quality of life in a large sample. J Psychiatr Res (2018) 96:145–52. 10.1016/j.jpsychires.2017.10.003 29049971

[17] Brunault P Courtois R Gearhardt A Gaillard P Journiac K Cathelain S Validation of the French Version of the DSM-5 Yale Food Addiction Scale in a Nonclinical Sample. Can J Psychiatry (2017) 62:199–210. 10.1177/0706743716673320 28212499 PMC5317020

[18] Meule A Müller A Gearhardt AN Blechert J German version of the Yale Food Addiction Scale 2.0: Prevalence and correlates of “food addiction” in students and obese individuals. Appetite (2017) 115:54–61. 10.1016/j.appet.2016.10.003 27717658

[19] Aloi M Rania M Muñoz RCR Murcia SJ Fernández-Aranda F De Fazio P Validation of the Italian version of the Yale Food Addiction Scale 2.0 (I-YFAS 2.0) in a sample of undergraduate students. Eat Weight Disord (2017) 22:527–33. 10.1007/s40519-017-0421-x 28780748

[20] Granero R Jiménez-Murcia S Gerhardt AN Agüera Z Aymamí N Gómez-Peña M Validation of the Spanish Version of the Yale Food Addiction Scale 2.0 (YFAS 2.0) and Clinical Correlates in a Sample of Eating Disorder, Gambling Disorder, and Healthy Control Participants. Front Psychiatry (2018) 9:208. 10.3389/fpsyt.2018.00321 29887808 PMC5980980

[21] Imperatori C Fabbricatore M Lester D Manzoni GM Castelnuovo G Raimondi G Psychometric properties of the modified Yale Food Addiction Scale Version 2.0 in an Italian non-clinical sample. Eat Weight Disord (2019) 24:37–45. 10.1007/s40519-018-0607-x 30414076

[22] Nunes-Neto PR Köhler CA Schuch FB Quevedo J Solmi M Murru A Psychometric properties of the modified Yale Food Addiction Scale 2.0 in a large Brazilian sample. Rev Bras Psiquiatr (2018) 40:444–8. 10.1590/1516-4446-2017-2432 PMC6899372 29898195

[23] Burrows T Kay-Lambkin F Pursey K Skinner J Dayas C Food addiction and associations with mental health symptoms: a systematic review with meta-analysis. J Hum Nutr Diet (2018) 31:544–72. 10.1111/jhn.12532 29368800

[24] Penzenstadler L Soares C Karila L Khazaal Y Systematic Review of Food Addiction as Measured With the Yale Food Addiction Scale: Implications for the Food Addiction Construct. Curr Neuropharmacol (2018) 17:526–38. 10.2174/1570159X16666181108093520 PMC6712300 30406740

[25] Ouellette AS Rodrigue C Lemieux S Tchernof A Biertho L Bégin C An examination of the mechanisms and personality traits underlying food addiction among individuals with severe obesity awaiting bariatric surgery. Eat Weight Disord (2017) 22:633–40. 10.1007/s40519-017-0440-7 29022218

[26] Meule A de Zwaan M Müller A Attentional and motor impulsivity interactively predict ‘food addiction’in obese individuals. Compr Psychiatry (2017) 72:83–7. 10.1016/j.comppsych.2016.10.001 27768944

[27] Clark SM Martens K Smith-Mason CE Hamann A Miller-Matero LR Validation of the Yale Food Addiction Scale 2.0 Among a Bariatric Surgery Population. Obes Surg (2019) 29:2923–8. 10.1007/s11695-019-03927-z 31119701

[28] Ivezaj V Wiedemann AA Grilo CM Food addiction and bariatric surgery: a systematic review of the literature. Obes Rev (2017) 18:1386–97. 10.1111/obr.12600 PMC5691599 28948684

[29] Naish KR MacKillop J Balodis IM The Concept of Food Addiction: a Review of the Current Evidence. Curr Behav Neurosci Rep (2018) 5:281–94. 10.1007/s40473-018-0169-2

[30] Müller A Leukefeld C Hase C Gruner-Labitzke K Mall JW Köhler H Food addiction and other addictive behaviours in bariatric surgery candidates. Eur Eat Disord Rev (2018) 26:585–96. 10.1002/erv.2629 30094889

[31] Bourdier L Orri M Carre A Gearhardt AN Romo L Dantzer C Are emotionally driven and addictive-like eating behaviors the missing links between psychological distress and greater body weight? Appetite (2017) 120:536–46. 10.1016/j.appet.2017.10.013 29030085

[32] Benzerouk F Gierski F Ducluzeau P-H Bourbao-Tournois C Gaubil-Kaladjian I Bertin É Food addiction, in obese patients seeking bariatric surgery, is associated with higher prevalence of current mood and anxiety disorders and past mood disorders. Psychiatry Res (2018) 267:473–9. 10.1016/j.psychres.2018.05.087 29980127

[33] Carlson L Steward T Agüera Z Mestre-Bach G Magaña P Granero R Associations of food addiction and nonsuicidal self-injury among women with an eating disorder: A common strategy for regulating emotions? Eur Eat Disord Rev (2018) 26:629–37. 10.1002/erv.2646 30318670

[34] Carter JC Van Wijk M Rowsell M Symptoms of ‘food addiction’ in binge eating disorder using the Yale Food Addiction Scale version 2.0. Appetite (2019) 133:362–9. 10.1016/j.appet.2018.11.032 30508614

[35] de Vries S-K Meule A Food Addiction and Bulimia Nervosa: New Data Based on the Yale Food Addiction Scale 2.0. Eur Eat Disord Rev (2016) 24:518–22. 10.1002/erv.2470 27578243

[36] Schulte EM Gearhardt AN Associations of Food Addiction in a Sample Recruited to Be Nationally Representative of the United States. Eur Eat Disord Rev (2018) 26:112–9. 10.1002/erv.2575 29266583

[37] Le Sphinx Mise en place d"une enquête avec Sphinx, support d"utilisation, service formations. Le Sphinx Développement: Annecy (FR) (2010). Available at: http://www.lesphinx.eu.

[38] Gormally J Black S Daston S Rardin D The assessment of binge eating severity among obese persons. Addict Behav (1982) 7:47–55. 10.1016/0306-4603(82)90024-7 7080884

[39] Brunault P Gaillard P Ballon N Couet C Isnard P Cook S [Validation of the French version of the Binge Eating Scale: examination of its factor structure, reliability, and convergent and divergent validity in a non clinical and a c clinical population]. Encephale (2016) 42:426–33. 10.1016/j.encep.2016.02.009 27017318

[40] Karlsson J Persson LO Sjöström L Sullivan M Psychometric properties and factor structure of the Three-Factor Eating Questionnaire (TFEQ) in obese men and women. Results from the Swedish Obese Subjects (SOS) study. Int J Obes Relat Metab Disord (2000) 24:1715–25. 10.1038/sj.ijo.0801442 11126230

[41] de Lauzon B Romon M Deschamps V Lafay L Borys J-M Karlsson J The Three-Factor Eating Questionnaire-R18 is able to distinguish among different eating patterns in a general population. J Nutr (2004) 134:2372–80. 10.1093/jn/134.9.2372 15333731

[42] R Development Core Team (2013) R: A language and environment for statistical computing. Vienna, Austria: R Foundation for Statistical Computing (2003). Consulted at: http://www.R-project.org [10-april 2019].

[43] Revelle W Psych: procedures for personality and psychological research. Version 1.3.10. Northwestern University: Evanston (IL) (2013). Available at: http://CRAN.R-project.org/package=psych.

[44] Muthén LK Muthén B Mplus. Mplus User"s Guide. Eight Edition Los Angeles, CA: Muthén & Muthén (2018).

[45] Kline RB Beyond significance testing: reforming data analysis methods in behavioral research. 1st ed Washington, DC: American Psychological Association (2004).

[46] Bentler PM On tests and indices for evaluating structural models. Pers Individ Differ (2007) 42:825–9. 10.1016/j.paid.2006.09.024

[47] Brown TA Confirmatory factor analysis for applied research. New York: Guilford Publications (2014).

[48] Dunn TJ Baguley T Brunsden V From alpha to omega: a practical solution to the pervasive problem of internal consistency estimation. Br J Psychol (2014) 105:399. 10.1111/bjop.12046 24844115

[49] Falissard B Mesurer la subjectivité en santé: perspective méthodologique et statistique. Elsevier Masson: Paris (2008).

[50] Kline P Handbook of Test Construction: Introduction to Psychometric Design. London: Taylor & Francis (2015).

[51] Beck AT Steer RA Carbin MG Psychometric properties of the Beck Depression Inventory: Twenty-five years of evaluation. Clin Psychol Rev (1988) 8:77–100. 10.1016/0272-7358(88)90050-5

[52] Collet L Cottraux J [The shortened Beck depression inventory (13 items). Study of the concurrent validity with the Hamilton scale and Widlöcher’s retardation scale]. Encephale (1986) 12:77–9. 3743520

[53] Meule A Food addiction and body-mass-index: A non-linear relationship. Med Hypotheses (2012) 79:508–11. 10.1016/j.mehy.2012.07.005 22854106

[54] Rozin P Kabnick K Pete E Fischler C Shields C The Ecology of Eating: Smaller Portion Sizes in France Than in the United States Help Explain the French Paradox. Psychol Sci (2003) 14:450–4. 10.1111/1467-9280.02452 12930475

[55] Pursey KM Stanwell P Gearhardt AN Collins CE Burrows TL The prevalence of food addiction as assessed by the Yale Food Addiction Scale: a systematic review. Nutrients (2014) 6:4552–90. 10.3390/nu6104552 PMC4210934 25338274

[56] Chao AM Shaw JA Pearl RL Alamuddin N Hopkins CM Bakizada ZM Prevalence and psychosocial correlates of food addiction in persons with obesity seeking weight reduction. Compr Psychiatry (2017) 73:97–104. 10.1016/j.comppsych.2016.11.009 27930952 PMC5269607

[57] Guerrero Pérez F Sánchez-González J Sánchez I Jiménez-Murcia S Granero R Simó-Servat A Food addiction and preoperative weight loss achievement in patients seeking bariatric surgery. Eur Eat Disord Rev (2018) 26:645–56. 10.1002/erv.2649 30353597

[58] Malik S Mitchell JE Engel S Crosby R Wonderlich S Psychopathology in bariatric surgery candidates: a review of studies using structured diagnostic interviews. Compr Psychiatry (2014) 55:248–59. 10.1016/j.comppsych.2013.08.021 PMC3985130 24290079

[59] Fernandez-Aranda F Karwautz A Treasure J Food addiction: A transdiagnostic construct of increasing interest. Eur Eat Disord Rev (2018) 26:536–40. 10.1002/erv.2645 30338592

[60] Price M Higgs S Lee M Self-reported eating traits: Underlying components of food responsivity and dietary restriction are positively related to BMI. Appetite (2015) 95:203–10. 10.1016/j.appet.2015.07.006 26162952

[61] Davis C A commentary on the associations among “food addiction”, binge eating disorder, and obesity: Overlapping conditions with idiosyncratic clinical features. Appetite (2017) 115:3–8. 10.1016/j.appet.2016.11.001 27816464

[62] Gearhardt AN White MA Masheb RM Morgan PT Crosby RD Grilo CM An examination of the food addiction construct in obese patients with binge eating disorder. Int J Eat Disord (2012) 45:657–63. 10.1002/eat.20957 PMC3375872 22684991

[63] Schulte EM Grilo CM Gearhardt AN Shared and unique mechanisms underlying binge eating disorder and addictive disorders. Clin Psychol Rev (2016) 44:125–39. 10.1016/j.cpr.2016.02.001 PMC5796407 26879210

[64] Hebebrand J Albayrak Ö Adan R Antel J Dieguez C de Jong J “Eating addiction”, rather than “food addiction”, better captures addictive-like eating behavior. Neurosci Biobehav Rev (2014) 47:295–306. 10.1016/j.neubiorev.2014.08.016 25205078

[65] Ruddock HK Christiansen P Halford JCG Hardman CA The development and validation of the Addiction-like Eating Behaviour Scale. Int J Obes 2005 (2017) 41:1710–7. 10.1038/ijo.2017.158 PMC5682562 28676680

[66] Tuomela J Kaprio J Sipilä PN Silventoinen K Wang X Ollikainen M Accuracy of self-reported anthropometric measures — Findings from the Finnish Twin Study. Obes Res Clin Pract (2019) 13:522–8. 10.1016/j.orcp.2019.10.006 PMC9234778 31761633

[67] Buchwald H Avidor Y Braunwald E Jensen MD Pories W Fahrbach K Bariatric surgery: a systematic review and meta-analysis. JAMA (2004) 292:1724–37. 10.1001/jama.292.14.1724 15479938

[68] Carr MM Catak PD Pejsa-Reitz MC Saules KK Gearhardt AN Measurement invariance of the Yale Food Addiction Scale 2.0 across gender and racial groups. Psychol Assess (2017) 29:1044–52. 10.1037/pas0000403 27893229

